# Therapeutic Strategies for Dry Mouth Management with Emphasis on Electrostimulation as a Treatment Option

**DOI:** 10.1155/2021/6043488

**Published:** 2021-10-22

**Authors:** Amela Tulek, Aida Mulic, Martine Hogset, Tor Paaske Utheim, Amer Sehic

**Affiliations:** ^1^Department of Oral Biology, Faculty of Dentistry, University of Oslo, Oslo, Norway; ^2^Nordic Institute of Dental Materials (NIOM), Oslo, Norway; ^3^Department of Maxillofacial Surgery, Oslo University Hospital Ullevaal, Oslo, Norway; ^4^Department of Medical Biochemistry, Oslo University Hospital, Oslo, Norway

## Abstract

**Objectives:**

Xerostomia is a subjective sensation of dry mouth. It is commonly associated with salivary gland hypofunction. Both changes in the composition of the saliva and a reduction in the quantity secreted may be an objective finding of dry mouth. Although there are no currently available cures for the conditions resulting in dry mouth, there are several treatment options that give hope for patients who suffer from xerostomia. Individuals with some residual salivary gland function, which are contraindicated to pharmacological therapies, would benefit the most from identifying novel, alternative effective methods for stimulating production of saliva. The aim of this study was to give an overview of the latest and most relevant data related to treatment modalities for the management of dry mouth conditions. *Data Resources and Study Selection.* The present review was prepared by searching the National Library of Medicine database using the relevant medical terms and their combinations. A total of thirty-three studies met the inclusion criteria. Data were extracted by one author and verified by another.

**Conclusion:**

A number of patients showed positive treatment outcomes, and the adverse effects of both electrical stimulation (ES) and acupuncture have been reported as mild and transient. In patients who have undergone radiotherapy, acupuncture is shown to increase salivation. However, in patients with Sjogren's syndrome, the effects of ES devices seem to be elusive. Moreover, due to the instability of the findings in relation to longevity of clinical effect, patient satisfaction, quality of life, and clinical effectiveness of such treatments, the results remain vague.

## 1. Saliva and Oral Implications

The multiple functions of saliva, which are linked to its specific components and fluid characteristics, are crucial for maintaining the hypotonic environment; remineralization of dental enamel; control of the composition of oral microflora with its antibacterial, antiviral, and antifungal properties; mastication and swallowing; digestion of food; articulation of speech; and many other functions [1–4]. Approximately 0.6 L of saliva is excreted daily by major and minor salivary glands [5, 6].

The secretion of salivary components is dependent upon the autonomic nervous system and regulated by several reflexes. The masticatory and gustatory afferent impulses, and thermoreceptive, olfactory, nociceptive, and psychic stimuli influence activation of the salivary gland cells [7–9]. These afferent sensory impulses are transmitted to the salivation center in the brainstem (parasympathetic), to the upper thoracic segments of the spinal cord (sympathetic), and to higher brain structures, which may react with both inhibitory and excitatory efferent signals to the salivatory nuclei and thereby salivary glands [10].

Furthermore, the function of salivary glands is under the influence of many stimuli and factors, which can affect the flow, volume, and composition of saliva. It has been shown that the expression of aquaporin 5 and of specific clock genes involved in the regulation of circadian rhythms is subject to diurnal pattern in gene expression in mouse submandibular gland cells [11]. Thus, it is suggested that the molecular underpinnings orchestrating normal salivary secretion may be influenced by the circadian clock, which also may play a role in different pathologies of salivary glands [12]. The size of the glands and the level of hydration of the body are other factors related to salivary flow rates [13–16].

From the clinical aspect, both changes in the composition of saliva and a reduction in the quantity secreted may be an objective finding of dry mouth [17, 18]. Dry mouth is a common problem in the general population with a prevalence between 10% and 33%, being more common in females [19, 20]. Although xerostomia more frequently affects the elderly, it may also be present in young adults [21, 22]. The subjective sensation of dry mouth in the older population may probably be attributed to other factors than age-related degenerative changes [23]. The higher prevalence of chronic conditions and the resultant general “polypharmacy” with wide use of various anticholinergic medications are considered as important factors [20, 24]. Several medications, including commonly prescribed preparations, such as those used to treat hypertension, epilepsy, and depression, are reported to cause dry mouth through different mechanisms [20, 24].

In general, causes of xerostomia can be divided into two main groups: nonsalivary and salivary [17]. Nonsalivary causes of oral dryness may include dehydration, anxiety, mouth breathing, and neurological dysfunction [17], whereas the salivary causes of dry mouth are associated with pathological changes in salivary glands. A number of patients with systemic autoimmune diseases such as Sjogren's syndrome, systemic lupus erythematosus, rheumatoid arthritis, systemic sclerosis, and sarcoidosis suffer from dry mouth [25, 26]. Exocrine glands in those patients become infiltrated with immune cells, predominantly CD4+ lymphocytes T-cells. The lymphocytes further induce the production of different cytokines that alter saliva secretion [27]. Simultaneously, an increased activity of certain matrix metalloproteinase, with a decrease in production of tissue inhibitors of such proteolytic enzymes, is found in those patients [28]. Disturbance of saliva production is also reported in patients with diabetes mellitus, obesity, hypertension, chronic kidney disease and chronic heart failure [29–34]. Common in those patients is increased predisposition to oxidative stress. Products of oxidation can aggregate and accumulate in the salivary glands leading to damage of secretory cells and increase in the formation of reactive oxygen species. This may enhance local oxidative stress even more [35–37].

A noteworthy correlation was found between salivary gland hypofunction, followed by xerostomia and patients suffering from dementia [38, 39]. One plausible explanation is that neurological centers orchestrating function of salivary glands are impaired in those patients. However, this field requires more research in order to draw affirmative conclusions.

Xerostomia was reported in majority of COVID-19 patients. Since the virus has neuropathic and mucotropic effects on the salivary gland tissues, it is hypothesized that it alternates the structure and function of the gland at some point. As xerostomia occurs prior to other common symptoms of infection, this information could be used as an early diagnostic mark [40].

Furthermore, dry mouth is a common side effect of both chemotherapy and radiotherapy during the treatment of cancer [20, 41].

Since the treatment of chronic diseases becomes more effective and the life expectancy increases, it is also reasonable to assume that the number of people living with xerostomia will be higher [20, 23]. Dry mouth conditions may impact the quality of life in several ways. This includes difficulties with speech, chewing, and swallowing of food, due to the dehydrated mucosa [42]. Taste sensation can be impaired and tenderness of the oral mucosa and gums makes the wearing of dentures difficult. Infections in salivary glands, oral candidiasis, and increased incidence of root caries have been reported [18, 43, 44]. A positive correlation between xerostomia and/or hyposalivation and caries activity in a population of younger adult patients has been shown recently [45]. Patients with subjective sensation of dry mouth complain of burning mouth syndrome [46]. Furthermore, psychological discomfort and impaired sleep may be another consequence. Finally, majority of patients can feel stigmatized, as their condition is often not taken seriously by others. This leads to social withdrawal and diminished self-esteem [42]. Effective treatment of patients with dry mouth is therefore important in order to improve the quality of life of sufferers, and, in addition, from both a patient and public health perspective it is of outmost importance to treat dry mouth symptoms and minimize potentially painful oral infections and costly tooth loss.

Since xerostomia may affect the quality of life in individuals, the main aim of this study was therefore to provide an overview of the latest and most relevant findings related to treatment modalities for the management of dry mouth conditions.

## 2. Data resources and Study Selection

Data collected from the National Library of Medicine database were used in this study. The search was conducted with the different combinations of the following terms: (“dry mouth” OR “xerostomia” OR “hyposalivation”) AND (“treatment”) AND (“electrical stimulation” OR “electrotherapy” OR “acupuncture”) in an attempt to reveal relevant publications. The search was performed without restriction with regard to the language and study design. Selection criteria included articles published from 1981 to the present year. Primary search yielded 187 studies. All authors thereafter reviewed the titles and abstracts of the selected articles. Eventually, duplicates were excluded. Articles were also rejected if they were clearly unqualified. In case where updated versions of the paper were found, older versions were rejected. The authors then reviewed the remaining articles to determine their eligibility. Finally, thirty-three studies were accessed.

### 2.1. Current Treatment Options

Although there are no currently available cures for the conditions resulting in dry mouth, different treatment options give hope for patients who suffer from xerostomia. The most commonly used approaches are the use of salivary substitutes and increased fluid intake, which aim to treat the symptoms. Some individuals may manage the problems associated with dry mouth through optimal handling of the underlying conditions [23]. For patients with milder symptoms, frequent sips of water and sucking of ice chips may result in sufficient relief [19]. It is recommended to reduce/avoid the consumption of alcoholic drinks, caffeine, and smoking as they additionally dehydrate the oral mucosa [47, 48]. Topical application of oral rehydrating agents acts directly on the surface of the oral mucosa and may provide short-term relief [24]. It has been shown that the use of oral moisturizers and toothpastes result in significant improvements in whole unstimulated salivary flow rate, a decrease in colonization with *Candida*, and a subjective improvement of xerostomia in patients with primary Sjogren's syndrome and subjects that had undergone radiotherapy for head and neck cancer [49, 50]. It is important to note that the utility of artificial saliva products is to some extent limited in xerostomia treatment, due to the different composition of artificial saliva compared to human saliva with respect to pH values, osmolality, and electrical conductivity [51].

The symptomatic treatment of xerostomia by means of topical medications stimulating saliva production or increased fluid intake may be sufficient for patients with some degree of preserved salivary gland function [20]. However, in patients with permanent destruction of salivary acini, in addition to palliative therapy, other forms of both local and systemic treatment may be necessary in order to induce secretions from the remaining salivary gland tissue. The two most common systemic agents are pilocarpine and cevimeline, which both act as the agonist for the muscarinic receptors on the surface of the salivary cells [52]. Several clinical studies have demonstrated that the use of pilocarpine results in significant improvement in symptoms associated with xerostomia [53–55]. However, since it is a cholinergic agent, it is associated with several side effects like increased sweating and lacrimation, frequent urination, nausea, headache, rhinitis, and gastrointestinal disturbances [56]. Cevimeline is an analog of acetylcholine and gives the same amount of relief from symptoms of dry mouth as pilocarpine. However, due to its high affinity to the M3 sub-receptor type, which is specific to the salivary gland tissue, it has fewer side effects compared to pilocarpine [52]. Bethanechol and anethole trithione are other drugs that exert their function via the parasympathetic system and have been shown to have some effect on dry mouth symptoms [52]. A study conducted on head and neck radiation therapy patients treated with either bethanechol or pilocarpine suggested that both medications have nearly the same effect on saliva production [57]. It is also worth mentioning another medicine, amifostine, currently the only medicine used in an attempt to prevent xerostomia in radiation therapy patients, due to its ability to scavenge free radicals [58].

Gene therapy in the treatment of dry mouth is based on delivery of genes into the salivary glands. Although it may become a therapeutic strategy for radiation-induced salivary hypofunction in the future, current therapies are primarily experimental, with most studies performed in animal models [59]. One clinical trial employing gene delivery to salivary gland in head and neck irradiated participants studied gene transfer utilizing the first-generation serotype 5, adenoviral (Ad5) vector coding for human aquaporin-1 (hAQP1) [60]. AQP1, expressed in the myoepithelial and endothelial cells of the human [61, 62], is a water channel protein that facilitates fluid transfer by an osmotic gradient [63]. Delivery of AdhAQP1 vector to a single parotid gland was found to be safe and the results demonstrated that the transfer of the hAQP1 cDNA increased the flow in parotid gland and relieved symptoms in a subset of patients [64]. The improvements in these patients persisted for several years after the one-time treatment. At present, there are two ongoing clinical trials evaluating the delivery of aquaporin-1 (AQP1) via adeno-associated viral vector 2 (AAV2) to the parotid gland in human patients with radiation-induced salivary gland hypofunction [65, 66]. Both studies are in phase 1 with the estimated study completion in 2022.

Another potential approach in regeneration of salivary gland tissue is the use of stem cells. Although clinical trials are mostly in its early phases, the obtained results in radiation-induced xerostomic patients seem to be promising [67]. The stem cells used in dry mouth treatment are mainly mesenchymal/stromal cells harvested from umbilical cord blood, bone marrow, or adipose tissue [68–71]. However, due to their potential to metastasize to other tissues, the long-term safety of these cells is yet to be investigated.

The quality of life of a patient suffering from radiation-induced xerostomia may be significantly reduced. Therefore, the use of low-power laser light for analgesive and anti-inflammatory effect has been suggested. The laser light that is transformed into energy for the cells improves microcirculation, glandular cell proliferation, cellular respiration, ATP production, protein synthesis, and intracellular calcium levels [72]. Having in mind that this technique is noninvasive and noncostly, it could be used in xerostomia treatment.

Intraglandular administration of botulinum toxin prior to radiation treatment is another promising approach in the treatment of radiation-induced xerostomia. Although the mechanism of action is not yet clear, it is suggested that botulinum toxin reduces nerve stimulation and saliva production, thereby reducing the sensitivity of glandular cells to radiation. However, agreements for the use of this technique are still in the establishment phase [73, 74].

### 2.2. Electrical Stimulation as a means for Treating Dry Mouth

Electrical activity is essential for the development, function, and survival of neurons [75]. Already in 1791, Luigi Galvani`s animal experiments demonstrated that the application of electrical current resulted in contractions in the muscles of frog legs. Since then, it has been known that electrical stimulation (ES) may be a common rehabilitative strategy to restore function in muscle and neural tissues. Cardioversion and defibrillation further illustrate the huge therapeutic potential of ES in medicine, and evolving research has provided evidence that ES of the eye may be a promising therapy for either preserving or restoring vision in several retinal and optic nerve diseases [76].

Electrostimulation of the salivary glands and acupuncture aim to increase the production of saliva. In ES, a hand-held battery-operated device is used to provide an electrical stimulus to the hard palate or dorsum of the tongue. Alternatively, a transcutaneous electrical nerve stimulation (TENS) device may be utilized by connecting the electrodes to the skin and may be used either in clinical setting or in the patient's home. Acupuncture and ES have been shown to exert both clinical and biological effect with regard to the treatment of dry mouth [44, 77]. It has been demonstrated that application of electrical impulses to one or more arms of the salivary reflex arch may result in increased secretion of saliva [43]. ES of the lingual nerve, i.e., the efferent trigeminal fibers, may stimulate the sublingual and submandibular glands to increase salivation [44]. Regarding acupuncture, it is suggested to induce physiological effects such as increased peripheral blood flow and stimulation of the autonomic nervous system, which in turn may lead to increased production of saliva [78].

The beneficial effects of noninvasive ES in patients with xerostomia due to Sjögren's syndrome and radiation therapy have been reported previously, which sparked a research interest in the effects of ES in clinical studies for treating dry mouth [79]. The methods include therapies such as acupuncture and acupuncture-like TENS [80], both of which focus on specific points in and around the head and neck region and aim to stimulate the parasympathetic innervation of salivary glands. The first study on acupuncture for the treatment of xerostomia was reported in 1981 [81], and since then, many studies using this method have reported an increase in salivary flow rate in both healthy and diseased patients (Table 1). Blom and collaborators showed that the effects of acupuncture resulted in relief for patients who have undergone head and neck radiation therapy and in individuals with Sjogren's syndrome. The study also demonstrated that the relief reported by the patients continued for up to six months posttreatment [86]. Another study reported that the use of acupuncture in patients with xerostomia increased the amount of calcitonin gene-related peptide in saliva [85], a protein known to positively affect the salivary flow and provide beneficial trophic effects on the oral mucosa. Three other controlled trials studied the therapeutic efficacy of acupuncture in patients that exhibited radiation-induced xerostomia [84, 90, 96]; however, they all exhibited the high risk of bias due to the overall poor reporting of blinding and randomization. A single-blinded study showed no significant difference in salivary flow in the active acupuncture group compared with control patients (sham acupuncture) after six weeks of twice-a-week acupuncture treatment [90]. Furthermore, Simcock et al. investigated the effects of eight weeks of once-a-week acupuncture compared to the effects of the use of artificial saliva, and they reported an improvement in xerostomia symptoms in acupuncture patients compared to controls [96]. However, in this study, the magnitude of the improvement, as well as clinical significance, was indistinct. No statistical difference in salivary flow between individuals randomized to twelve weeks of real or sham acupuncture was also reported by Blom et al. in 1996 [84]. A recent study from 2019, a randomized clinical trial, found that true acupuncture resulted in significantly fewer and less severe symptoms one year after treatment vs standard care control and sham acupuncture in radiation-induced xerostomia patients, although authors reported inconsistence in results with sham acupuncture and suggested further research to confirm their findings clinically [114].

Acupuncture-like TENS perform the same objective as acupuncture without using the needles and has been shown to be effective. Wong and colleagues utilized a method termed *Codetron* to deliver electric nerve stimulation twice a week for six weeks to patients with symptomatic xerostomia resulting from radiation therapy [87]. They evaluated the residual salivary function and the results demonstrated statistically significant improvements in both subjective complaints of dry mouth and salivary flow rates for up to six months after treatment [81]. Another study investigated the effects of twelve weeks of acupuncture-like TENS (twice per week for a total of 24 sessions of 20 min each) versus pilocarpine and showed no significant difference in salivary flow or xerostomia-related quality of life scale score between the groups [102]. However, this study lacked the detailed information regarding the blinding, sequence generation, and allocation concealment.

## 3. Conclusion and Future Perspectives

Xerostomia, a symptom of dry mouth, is a condition that at present has no definitive means for treatment. Several inductive and palliative treatment approaches appear to be effective for reducing the morbidity associated with xerostomia. Most of the available treatment options are quite simply transient and are not considered to be an optimal treatment option. The present review has its limitations. The studies included in the work were reported between 1981 and 2021 and the patient population has changed during this time. Furthermore, the radiotherapy modalities have also progressed, where patients included in the most recent studies receive lower radiation dosage to salivary tissues.

**Figure 1:**
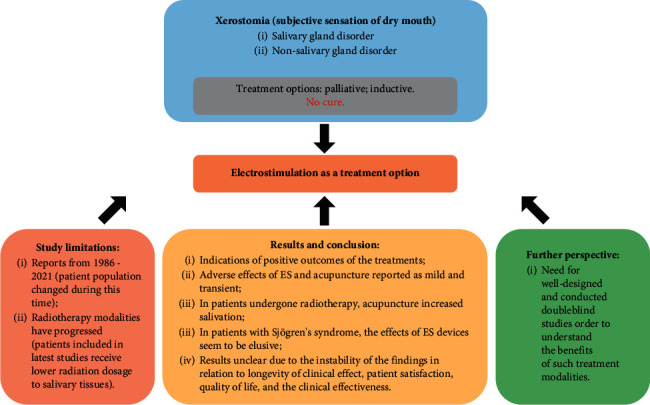
Main conclusions of the study: schematic representation.

So far, there is poor evidence on the effects of any of the interventions included in this review on patients with xerostomia. A number of patients with xerostomia show some indications of positive outcomes of these treatments, and the adverse effects of both ES and acupuncture have been reported as mild and transient. In patients who have undergone radiotherapy, the acupuncture is shown to increase salivation. However, in patients with Sjogren's syndrome, the effects of ES devices seem to be elusive. Moreover, due to the instability of the findings in relation to longevity of clinical effect, patient satisfaction, quality of life, and the clinical effectiveness of such treatments, the results remain unclear. Further well-designed and conducted double-blind studies are warranted in order to understand the benefits of these treatment modalities (Figure 1).

## Figures and Tables

**Table 1:** Electrical stimulation for treating dry mouth in human trials.

Reference	Disease	Stimulation location and parameters	Control group	Evaluation method	Results/conclusion
Weiss et al. [79]	24 patients in 3 groups (1) Sjögren´s syndrome, *N* = 9 (2) Undergone head and neck radiation therapy, *N* = 13 (3) Xerostomia being either drug-induced or unknown etiology, *N* = 3	Electrodes placed on the dorsum of the tongue and pressed against the hard palate; the maximum voltage was 6 V with a current of 9 uA; the maximum power was 12.4 mJ in a 3 minute stimulus cycle; the stimulus was increased until the patients reached their level of tolerance.	—	Subjective patient evaluation was performed Intraoral clinical examination was performed.	This noninvasive electrostimulation has shown to be effective in patients with xerostomia due to Sjögren's syndrome or radiation therapy; patients reported slight to substantial improvement.

Steller et al. [82]	Sjögren´s syndrome, *N* = 29	Electrodes placed on the dorsum of the tongue and pressed against the hard palate. An intensity control knob with intervals from 0 to 10 set peak stimulus output between 0 and 6 V; the maximum average current output is 9 uA and corresponds to a maximum average power dissipation of 0.2 uW.	Placebo device and electrodes	Subjective patient evaluation was performed. Intraoral clinical examination was performed The whole saliva flow rates were measured.	The mean poststimulation whole saliva flow rate of subjects using the active device increased, while the mean poststimulation rate of those using a placebo device did not (*P*=0.04). The results in this study indicate that some Sjögren's syndrome patients with residual salivary flow show a significant response to electrical stimulation, but others with low or absent whole saliva flow rates do not respond.

Talal et al. [83]	Sjögren´s syndrome, *N* = 77	Electrodes placed on the dorsum of the tongue and pressed against the hard palate. The unit stimulus switch was pressed, and the stimulus intensity was adjusted to settings of 1–10 with the objective of reaching a setting of 10. The stimulation automatically shut off after 3 min.	Placebo device and electrodes	The whole saliva flow rates were measured. The weight of the saliva was measured.	The patients using active devices showed a statistically greater (*P*=0.005 to 0.02) increase in the production of saliva compared to control. At week 1, the differences between the prestimulation and poststimulation salivary production was 80% greater in the group with the active device than in the control group; the differences were 151% at week 2 and 116% at week 4.
Blom et al. [84]	Undergone head and neck radiation therapy, *N* = 38	Classical acupuncture was used.	Classical acupuncture and superficial acupuncture as placebo		Both groups showed significantly increased salivary flow rates after the acupuncture treatment. In the experimental group 68% and in the control group 50% of the patients had increased salivary flow rates at the end of the observation period. Observation period of 1 year.

Dawidson et al. [85]	Healthy subjects, *N* = 8	Manual acupuncture and acupuncture with low-frequency electrical stimulation (2 Hz) were used.	—	Immunoreactivity of neuropeptides was analyzed in the saliva collected 20 minutes before the start of acupuncture stimulation, then for 20 minutes while the needles were in situ, and the for another 20 minutes after the needles were removed.	The results showed significant increases in the release of CGRP, NPY, and VIP both during and after acupuncture stimulation, especially in connection with electro-acupuncture.

Blom et al. [86]	Sjogren's syndrome, irradiation, and other causes, *N* = 70	Acupuncture and two-way ANOVA were used.	—	Salivary flow rates (SFR) for whole unstimulated and stimulated saliva were used as indicators of effects. Data were analyzed 6 month following a baseline course of 24 acupuncture treatments using two-way ANOVA.	Statistically significant differences in unstimulated and stimulated salivary flow rates (*P* < 0.01) in all etiological groups after 24 acupuncture treatments and up to 6 months follow-up compared with baseline.

Wong et al. [87]	Undergone head and neck radiation therapy, *N* = 46	Codetron treatment was given twice weekly for 6 weeks. Nonpolarizing, balanced biphasic, square electrical pulses of 250 ms duration were delivered in trains with a repetition rate of 4 hz. Each acupuncture point was randomly stimulated for 10 s each time; each session of codetron treatment lasted for a total of 20 min; this was followed by a 2-week break and then another 6-week course of treatment was repeated.	—	The whole saliva flow rates were measured. The quality of life questionnaire assessments were performed.	Improvement in xerostomia symptoms was noted, with a mean increase in the visual analog scale score of 86 (*P* < 0.0005) and 77 (*P* < 0.0001) at 3 and 6 months after treatment completion, respectively. For all patients, the increase in the mean basal and citric acid-primed whole saliva production at 3 and 6 months after treatment completion was also statistically significant (*P* < 0.001 and *P* < 0.0001, respectively). No statistically significant change in the quality-of-life evaluation compared with baseline was observed.
Hargitai et al. [88]	Healthy subjects, *N* = 22	The TENS electrode pads were placed externally on the skin overlying parotid glands. The pulse rate was fixed at 50 Hz, the pulse duration 250 *μ*sec, and the unit was in normal mode.	—	Unstimulated saliva was collected for 5 minutes via the carlson-crittenden cup placed over Stensen's duct bilaterally, into preweighted vials. The TENS unit was then activated, and stimulated saliva collected for an additional 5 minutes.	15 of 22 subjects demonstrated increased parotid salivary flow when stimulated via the TENS unit. Five experienced no increase and 2 experienced a decrease. The mean unstimulated salivary flow rates were 0.02418 mL/min and mean stimulated salivary flow rate was 0.04946 mL/min; the TENS unit was effective in increasing parotid gland salivary flow in two-third of healthy adult subjects.

Strietzel et al. [89]	Subjects with xerostomia, *N* = 23	Electrostimulation device named GenNarino; delivered during 10 min to the oral mucosa, in the mandibular third molar region (close to the area where the lingual nerve travels alongside the lingual alveolar plate).	Sham (inactive stimulation)	The digital signal of the wetness sensor expressed in numbers was used as a measure of dryness.	After 3 min of the 5 min experiments, significantly lower dryness was seen in the active modes compared with sham. No significant side effects were observed.

Cho et al. [90]	Undergone head and neck radiation therapy, *N* = 12	Acupuncture was conducted twice weekly for 6 weeks in a single-blind setting.	Randomized into two groups: Real or sham acupuncture	The effect was evaluated by measuring whole salivary flow rates and questionnaire-based assessment of subjective symptoms pretreatment and posttreatment (3 and 6 weeks after acupuncture treatment).	Both groups showed a slight increase in whole salivary flow rates, with no significant differences between them. Real acupuncture markedly increased unstimulated salivary flow rates, and improved the score for dry mouth according to the xerostomia questionnaire, by 2.33 points versus 0.33 in the controls.

Ami and Wolff [91]	Dry and burning mouth, *N* = 1	A Saliwell Crown was placed in the lower third molar area, in vicinity to the lingual nerve. The stimulating device is mounted on a commercially available dental implant. The Saliwell Crown is composed of an electric circuit, two 1.5 V batteries, a microprocessor, a wetness sensor, an IR receiver, and stimulating electrodes.	—	Both stimulated and unstimulated saliva flow rates were measured. Subjective patient evaluation was performed.	The results showed a constant significant increase in the salivary secretion and symptomatic improvement as presented in the questionnaires. The frequency of dry and burning mouth has decreased during the study.
Pattani et al. [92]	Undergone head and neck radiation therapy, *N* = 5	20 one-hour sessions with TENS unit; electrode pads placed externally on the skin overlying the parotid glands bilaterally; stimulation was initiated using 4 to 30 mA at 80–100 impulses. The settings ranged from 55 to 80 mA (maximum setting). The duration of treatment was 60 minutes per session. Patients received three E-stimulation treatments per week for a total of one to two months.	—	The whole saliva flow rates were measured. Dysphagia and xerostomia index questionnaire was used.	All five patients noticed a significant improvement (*P* value: 0.002) in dysphagia and a definite increase in saliva production. No untoward side effects/complications were noted in the patients during or after completion of treatment.

Wong et al. [93]	Undergone head and neck radiation therapy, *N* = 48	ALTENS treatments were administrated with a codetron transcutaneous electrical nerve stimulation (TENS) unit and karaya electrode pads. The locations of the acupuncture points used large intestine 4 (LI4), spleen 6 (SP6), stomach 36 (ST36), and conception vessel 24 (CV24). Square pulses of 250-msec duration were delivered in trains with a 4 hz repetition rate. One acupuncture point was stimulated for 10 seconds at a time. Random switching among electrodes was used. All patients received twice weekly ALTENS sessions (with 20 minutes of stimulation per session) for a total of 24 sessions given in 12 weeks. A maximum of 2 weeks without treatment was allowed, and all outstanding sessions were administrated.	—	Preliminary efficacy of ALTENS treatment was assessed using the patient-reported University of Michigan Xerostomia-Related Quality-of-Life-Scale (XeQOLS). The test was administrated at baseline and 6 months after study enrollment.	47 patients were evaluable. 44 of these 47 patients (94%) were successful in complying with ALTENS treatments. Of these, 34 patients (72%) completed all treatment sessions. Six-month XeQOLS were available for 35 patients and indicated that 30 patients (86%) achieved a positive treatment response with a mean ± standard deviation reduction of 35)±36,1%. There were no significant acute side effects associated with ALTENS treatment.
					The results indicated that ALTENS treatment for radiation-induced xerostomia can be delivered uniformly in a cooperative, multicenter setting and procedures with possible beneficial treatment response.

Strietzel et al. [94]	Sjögren's syndrome, *N* = 114	The electrodes directly contact the oral mucosa in the mandibular third molar area, in proximity to the lingual nerve.	Sham (inactive stimulation)	Subjective patient evaluation was performed. Intraoral clinical examination was performed. The whole saliva flow rates were measured.	The active device performed better than the sham device for patient-reported xerostomia severity (*P* < 0.002), xerostomia frequency (*P* < 0.05), quality of life impairment (*P* < 0.01), and swallowing difficulty (*P* < 0.02). At the end of stage 2, statistically significant improvements were verified for patient-reported xerostomia severity (*P* < 0.0001), xerostomia frequency (*P* < 0.0001), oral discomfort (*P* < 0.001), speech difficulty (*P* < 0.02), sleeping difficulty (*P* < 0.001), and resting salivary flow rate (*P* < 0.01).

Alajbeg et al. [95]	Xerostomia, *N* = 94	GenNarino containing an electronic circuit with a microprocessor, a pair of stimulating electrodes, and a 30 mA/h battery. The electrodes contact the oral mucosa in the mandibular third molar area, close to the lingual nerve. The electrical current is of low intensity and not felt by the patient.	—	Subjective patient evaluation was performed. Intraoral clinical examination was performed. The whole saliva flow rates were measured.	Improvements achieved at month 5 from baseline were sustained throughout the follow-up period for the primary outcome, xerostomia severity, and the second outcomes resting whole saliva flow rate, xerostomia frequency, oral discomfort and difficulties in speech, swallowing and sleeping. No significant side effects were detected.

Simcock et al. [96]	Undergone head and neck radiation therapy, *N* = 145	Randomized crossover design with participants receiving two group sessions of oral care education and eight acupunctures using standardized methods. Patient-reported outcome measures were completed at baseline and weeks 5, 9, 13, 17, and 21.	Acupuncture group and oral care education group	The whole saliva flow rates were measured.	Acupuncture compared with oral care, produced significant reductions in patients' reports of severe dry mouth with sticky saliva, needing to sip fluids to swallow food and in waking up at night to drink. No significant changes in either stimulated or unstimulated saliva measurements over time.

Pattipati et al. [97]	Healthy subjects, *N* = 90;	Transcutaneous electric nerve stimulation (TENS) placement of pads was approximated bilaterally over the parotid glands. The working parameters of TENS unit were fixed at 50 Hz and 250 µs pulse rate, and the unit was in normal mode.	—	The whole saliva flow rates were measured.	Subjects belonging to group B were showing a statistically significant increase in the duration of stimulated parotid salivary flow following the use of TENS.
	The study was carried out in three different age groups				
	a) Individuals from 21 to 35 years				
	b) 36–50 years				
	c) Above 51 years				
	In each group, 30 subjects were taken of whom 15 were males and 15 were females				

Vijayan et al. [98]	Undergone head and neck radiation therapy, *N* = 30	The transcutaneous electrical nerve stimulation (TENS) electrode pads were placed externally on the skin overlying the parotid glands using an extra-oral device, 1 cm in front of the tragus of the ear, over the parotid region with the TENS unit in the off position. The TENS machine used for the study was MEDIHIGHTEC 8000 combo (MEDIHIGHTEC medical company. Ltd., Taiwan). The pulse rate was fixed at 50 Hz, the pulse duration was fixed at 250 *μ*m, the time was fixed for 5 min, and the unit was used in normal mode.	—	Both stimulated and unstimulated saliva flow rates were measured.	29 of 30 patients showed increased saliva flow during stimulation. A statistically significant improvement in saliva production (*P* < 0.05) during stimulation was noted. Extra-oral application of TENS is effective in increasing the whole salivary flow in most of the postradiated oral cancer patients with xerostomia. TENS therapy may be useful as an effective supportive treatment modality in postradiated oral cancer patients.

Zadik et al. [99]	Oral chronic graft-versus-host disease (cGVHD), *N* = 6	The electrodes directly contact the oral mucosa in the mandibular third molar area, in proximity to the lingual nerve.	—	Clinical examination was performed. Both stimulated and unstimulated saliva flow rates were measured.	Two patients developed mild mucosal lesions in areas in contact with the GenNarino. However, only one of them had a change in the national institutes of health (NIH) score for oral cGVHD. The unstimulated and stimulated salivary flow increased in 4 out of the 5 patients included in the analysis.
Aggarwal et al. [100]	Healthy subjects, *N* = 80	The TENS unit was anlaya MedIns – AMS-902. The surface electrode pads were placed externally on the skin, overlying the parotid glands. The unit was preset at a frequency of 100 Hz and a pulse width of 100–150 *μ*s between the measurement of unstimulated and stimulated saliva. Then, the TENS unit was activated and the amplitude was gradually increased to a maximum tolerable level for patients.	—	Both stimulated and unstimulated saliva flow rates were measured.	The mean unstimulated flow rate was 1.25 ml/min, the mean stimulated salivary flow rate was 1,41 ml/min. 65 of 80 subjects demonstrate an increase in salivary flow rate on application of TENS. 12 subjects showed no increase in the salivation, while three subjects showed a decrease in the salivary flow. These findings showed an approximately 13% (0.16 ml/min) increase in the mean salivary flow rate on application of TENS.

Lakshman et al. [101]	Undergone head and neck radiation therapy, *N* = 40	TENS model-NS electro pulse that generates current through AC at a continuous frequency of 500 Hz with a sweep of 0.5–2 Hz. The electrodes are placed externally on the skin overlying the parotid glands.	Healthy subjects, *N* = 10	Both stimulated and unstimulated saliva flow rates were measured.	The data analysis revealed that control and S1B group showed increased salivary flow rate after stimulation by TENS therapy, compared with the unstimulated salivary flow, whereas in S1A and S1B group it was found to be statistically nonsignificant.

Wong et al. [102]	Undergone head and neck radiation therapy, *N* = 148	ALTENS were administrated with a codetron TENS unit and karaya electrode pads. Bilateral acupuncture points – SP6, ST36, LI4 – using uncommon electrodes and CV24 using the common electrode were stimulated. Sequences of 250 millisecond square pulses with a 4 Hz repetition rate were delivered. Each acupuncture point, except CV24, was stimulated for 10 seconds at a time; CV24, the site for the common electrode, was stimulated throughout the treatment session. Stimulation intensity was adjusted to produce a deep strong aching sensation at each acupuncture point. All patients were to receive 24 ALTENS sessions (20 minutes each, 2 sessions per week), for 12 weeks.	Randomized study that compared ALTENS with prilocarpine (PC) for relieving radiation-induced xerostomia. Patients: (73 in the pilocarpine group and 75 in the ALTENS group)	The primary endpoint was the change in the University of Michigan Xerostomia-Related Quality of Life scale (XeQOLS) scores from baseline to 9 months from randomization (MFR). Secondary endpoints included basal and citric-acid-primed whole salivary production (WSP), ratios of positive responders (defined as patients with 20% reduction in overall radiation-induced xerostomia symptom burden), and the presence of adverse events based on the common terminology criteria for adverse events version 3. An intention-to-treat analysis was conducted.	Only 96 patients completed the required XeQOLS and were evaluable at 9 MFR (representing merely 68.6% statistical power). Seventy-six patients were evaluable at 15 MFR. The median change in the overall XeQOLS in ALTENS and PC groups at 9 and 15 MFR were 0.53 and 0.27 (PZ.45) and 0.6 and 0.47 (PZ.21). The corresponding percentages of positive responders were 81% and 72% (PZ.34) and 83% and 63% (PZ.04). Changes in WSP were not significantly different between the groups. Grade 3 or less adverse events, mostly consisting of grade 1, developed in 20.8% of patients in the ALTENS group and in 61.6% of the PC group.
		Pilocarpine treatment started within 14 days of enrollment. Patient received 5 mg pilocarpine orally 3 times daily for 12 weeks and then stopped for the rest of the study.			

Hasegawa et al. [103]	Three groups: 20 young adults, 19 older adults, and 21 patients with dry mouth, *N* = 60	Used IFCS with a beat frequency of 50 Hz and a carrier frequency of 2000 Hz. The signal type was bipolar. Two independent pairs of electrodes were placed at bilateral submandibular regions symmetrically, targeting both the submandibular and sublingual glands.	—	Secreted saliva was collected by using salivette cotton rolls for 15 minutes, either with or without interferential current stimulation (IFCS). Patients were randomly chosen to receive IFCS. Each subject rated pain and discomfort on the visual analog scale (VAS) after each experiment. Saliva chromograin A levels were measured as a stress marker. To compare data between conditions with or without IFCS, a two-sample Student's *t*-test analysis was performed.	Saliva flow was slightly increased in those in the dry mouth group receiving IFCS compared with those who did not receive IFCS (approximately 130%). However, no such difference was found in the young and older adult groups. There was no significant difference in the VAS values of pain and discomfort or in the stress marker levels between patient who received or did not receive IFCS in the three groups. IFCS delivered to submandibular and sublingual glands may promote saliva secretion in persons who suffer from dry mouth in a manner that does not induce pain or physical stress.

Konidena et al. [104]	50 postmenopausal females with or without dryness Divided into 2 groups of 25 each; (1) postmenopausal women with oral dryness and (2) postmenopausal women without oral dryness	The TENS unit was ultrasonic TENS. The technical specifications of the TENS unit were 220 V, A/C 50 Hz, 0–100 mA at 1 k load, biphasic wave form, available in pulsed/continuous form, and 2 intensities, I and II. The electrode of TENS unit was then placed vertically, externally on the skin overlying the parotid gland, in the preauricular area bilaterally, 1 cm in front of the tragus area.	—	Unstimulated whole saliva was collected by the low forced splitting method. External salivary secretion of parotid glands by electrodes of TENS unit was done and sialometry was repeated. The salivary flow rates were compared within both groups before and after stimulation and between two groups.	The mean salivary flow rates at baseline were statistically significantly lower in group 1 than group 2. There was a mean increase of 0.33 ml and 0.46 ml with TENS stimulation in the two groups, respectively. Postmenopausal women with perception of oral dryness had lower salivary flow rates. 90% of the subjects, irrespective of oral dryness status, responded to TENS therapy. TENS stimulation resulted in a statistically significant increase in the quantity of whole saliva flow rate in postmenopausal women with or without dryness.

Aparna et al. [105]	Subjects with complaint of hyposalivation, *N* = 25	TENS units were placed over the parotid region. The intensity control switch was adjusted for patient's comfort. The intensity was turned up 1 increment at a time at 5 s intervals until the optimal intensity level was reached.	—	Unstimulated saliva was collected using modified carlson crittenden cup placed over the Stenson's duct bilaterally for 5 min and measured. A paired *t*-test, evaluating mean changes in stimulated versus unstimulated salivary flow rates.	Significant increase in parotid salivary flow rates (SFR) in 19 of 25 patients after TENS. Males showed more salivary secretion compared to females. TENS showed to be effective in increasing the SF rate in hyposalivatory patients with residual saliva.

Dyasnoor et al. [106]	Diabetes mellitus patients with xerostomia, *N* = 40	Tabletop TENS unit with surface electrode pads were placed externally in the skin overlying the parotid glands (anteroposteriorly between the tragus of the ear and the midmasseter region and superoinferiorly between the region of the head of the mandible and above the lower boarder of the mandible. The pulse rate was fixed at 50 Hz, and the intensity was gradually increased to a maximum tolerable level for each patient.	—	Unstimulated saliva through the “low forced splitting” method and stimulated saliva collecting using TENS were assessed and compared. Long-term effects of TENS application were evaluated by recalling the patient 24 hours later.	A statistically significant increase in stimulated whole saliva after TENS application in continuous mode (*P* < 0.001) was demonstrated compared with the unstimulated saliva, especially in xerostomia patients with diabetes. Burst mode inferred a statistically significant decrease in salivary flow (*P* < 0.001). In patients with diabetes with xerostomia and hyposalivation, TENS was highly effective in stimulating whole salivary flow.

Paim et al. [107]	Undergone head and neck radiation therapy, *N* = 15	TENS was adjusted with 50 Hz of frequency and 250 *μ*s of pulse width intensity was adjusted over a 20-minute period according to maximum tolerance. The electrodes were placed bilaterally on the region of the salivary glands.	—	Evaluation of the salivary flow was performed through sialometry before and immediately after application of TENS.	The most prevalent region for RT was the oropharynx (80% of cases). The mean dose used in RT was 64,6 ± 7,24 Gy. After TENS, salivary flow increased significantly (*P*=0.0051) from 0.05 mL/min to 0.01 mL/min. The response to TENS was directly correlated with the intensity of the tolerated electric current (*r* = 0.553; *P*=0.032) and the dose used in RT (*r* = −0.514; *P*=0.050). TENS was able to increase the salivary flow rate of patients with RT-induced hyposalivation.

Wolff et al. [108]	Severe xerostomia, no collectable saliva, *N* = 3	GenNarino contains an electronic circuit with a microprocessor, a pair of stimulation electrodes, and two 3 V 30 mAmp/h batteries. The electrodes contact the oral mucosa in the mandibular third molar area, close to the lingual nerve on one side. The electrical stimulation is of low intensity and not felt by the patient. In this trial, the stimulation signals were pulse-trains at 5 Hz, biphasic, at rectangular pulses of 1 mSec, with an output of 150 µA.	—	The whole saliva flow rates were measured.	All the three subjects developed the capacity to spit saliva, not only in direct response to the electrostimulation but also after free intervals without electrostimulation. In addition, their symptoms of dry mouth severity and frequency improved. For all three subjects, no significant changes in the vital signs and in the oral mucosal status were observed.

Yang et al. [109]	Hemodialysis patients, *N* = 80	250 *μ*s and 50 Hz TENS program were used.	Control group received a 50 *μ*s, 2 Hz TENS at acupoints ST6 and TE17 three times a week for 3 weeks	Whole salivary flow rate and dry mouth intensity were measured totally five times for both groups, at pretreatment, after 3, 6, and 9 TENS sessions, and one week after the treatment was completed.	After 6 TENS sessions were completed, whole salivary flow rates increased stably until the end of 9 TENS sessions for the treatment group. In the follow-up week after treatment, there was significant increase as well. However, significant improvement in dry mouth intensity was observed at all posttests than that at pretreatment in both groups. Whole salivary flow rates and dry mouth intensity improving were only observed during and one week after the TENS sessions.

Paim et al. [110]	Patients with hyposalivation, *N* = 68	TENS group (*n* = 37) received 8 sessions (20 minutes each) delivered twice a week for four weeks. The electrodes were attached over the skin covering the salivary glands. The electric pulse was adjusted at a frequency of 50 hz, pulse width of 250 *μ*s. and as intense as tolerated.	Participants in the control group (*N* = 31) received habitual care	SSF was evaluated through sialometry. Self‐perception of salivary flow (SPSF) and quality of life (QL) was evaluated prior to, during, and at 1, 3, and 6 months after treatment.	Although no changes were observed in the control group for SSF at any timepoints, TENS group showed a progressive increase in SSF from the third session until the end of the treatment. Significant improvements were also found in SPSF, especially when the SSF reached values ≥ 0.7 mL/minute. The most expressive results were evident at 6 months after treatment so that SSF, SPSF, and QL remained significantly higher (*F* = 9.5, *P*=0.0001; *H* = 143.77, *P* < 0.0001; *χ*2 = 9.162, *P*=0.02, respectively). TENS was effective at improving hyposalivation.

Ali et al. [111]	Diabetes mellitus type 2 patients with hyposalivation, *N* = 100, age range from 43 to 76 years	Randomly distributed to one experimental group, receiving one extra-oral 5 minutes TENS session, applied bilaterally on skin over parotid gland with frequency 50 Hz and pulse duration 250 *μ*s.	—	Unstimulated saliva was collected for 5 minutes immediately after the session, using the low forced spitting method. Stimulated saliva was collected during TENS application in graduated test tube.	90 out of 100 patients responded positively to TENS by increasing salivary volume and flow rate.

Iovoli et al. 2020 [112]	Patients treated with radiotherapy with or without chemotherapy for head and neck cancer (*N* = 30), with at least grade 1 or 2 symptomatic dry mouth	Patients randomly assigned to receive ALTENS four-times weekly for 6 weeks or two times weekly for 12 weeks.	—	XeQoLS was administered at 6, 9, 15, and 21 months from the start of ALTENS treatment. A linear model was used to model the overall XeQoLS score at the 15-month endpoint.	This study demonstrates that both ALTENS regimens are safe, well tolerated, and appear to be equally effective.

Koike et al. [113]	Healthy adults (*N* = 20)	Electrical stimulation was applied at constant strength for 60 minutes to the suprahyoid muscles using VitalStim®.	—	Participants examined under three conditions of NMES: Sensory threshold plus 75% of the difference between sensory and pain thresholds (75% stim), SensoryStim, and sham. Saliva collections, using a 10-min spitting method, were performed seven times: Before stimulation (S1), during stimulation (S2–S6), and 5 min after stimulation ended (S7).	Increase in saliva flow was promoted after NMES. Therefore, NMES may have effects on patients with xerostomia.

## Abbreviations

TENS: Transcutaneous electrical nerve stimulation
RT: Radiotherapy
SFR: Salivary flow rate
IR: Infrared
ALTENS: Acupuncture-like transcutaneous electrical nerve stimulation
VAS: Visual analog scale
SSF: Stimulated salivary flow
SPSF: Self‐perception of salivary flow
QL: Quality of life
XeQoLS: Xerostomia-related quality-of-life scale
cGVHD: Chronic graft-versus-host-disease
PC: Pilocarpine
WSP: Whole salivary production
MFR: Months from randomization
IFCS: Interferential current stimulation
LI4: Large intestine 4
SP6: Spleen 6
ST36: Stomach 36
CV24: Conception vessel 24
NMES: Neuromuscular electrical stimulation.
